# Checkpoint Inhibition for Metastatic Urothelial Carcinoma After Chemotherapy—Real-World Clinical Impressions and Comparative Review of the Literature

**DOI:** 10.3389/fonc.2020.00808

**Published:** 2020-05-21

**Authors:** Christian Fuhrmann, Julian P. Struck, Philipp Ivanyi, Mario W. Kramer, Marie C. Hupe, Bennet Hensen, Alexander Fürschke, Inga Peters, Axel S. Merseburger, Markus A. Kuczyk, Christoph-A. J. von Klot

**Affiliations:** ^1^Clinic for Urology and Urological Oncology, Hanover Medical School, Hanover, Germany; ^2^Department of Urology, University Hospital Schleswig-Holstein, Luebeck, Luebeck, Germany; ^3^Department of Hematology, Hemostasis, Oncology and Stem Cell Transplantation, Hanover Medical School, Hanover, Germany; ^4^Institute of Diagnostic and Interventional Radiology, Hanover Medical School, Hanover, Germany; ^5^Clinic for Radiology and Nuclear Medicine, University Hospital Schleswig-Holstein, Luebeck, Luebeck, Germany

**Keywords:** metastatic urothelial carcinoma, checkpoint inhibition, immunotherapy, atezolizumab, pembrolizumab, nivolumab

## Abstract

**Background:** The introduction of checkpoint inhibitors is a long-awaited new option for a urothelial cancer with a poor prognosis. Apart from clinical studies, the data on real world experience is scarce.

**Methods:** Patients for monotherapy with either Atezolizumab, Nivolumab or Pembrolizumab after chemotherapy were included. Adverse events and immune related adverse events as well as survival data and imaging analyses were recorded in a prospectively designed multi-center data base. Duration of response, progression free survival (PFS), and overall survival (OS) were estimated with the Kaplan-Meier method.

**Results:** A total of 28 patients were included. The median follow-up was 8.0 (range, 0.7–41.7) months. Median PFS was 5.8 (95% CI, 2.3–NA) months. Median OS for all patients was 10.0 (95% CI, 8.0–NA) months. The overall response rate (ORR) was 21.4% (6 out of 28 patients). Adverse events were recorded in 20 (71.4%) of patients. Higher grade adverse events (≥Grade 3) were present in 11 (39.3%) patients. No therapy related deaths occurred during the observation period. A total of 13 (46.4%) patients had adverse events that were considered to be immune related. The most commonly affected organ was the thyroid gland with 21.4% of events.

**Conclusion:** Our real-world clinical series confirms an objective response for about every fifth patient, promising OS and a low incidence for severe adverse events (≥Grade 3).

## Introduction

In Europe, ~151,000 new cases of urothelial carcinoma are diagnosed every year (1). Urothelial carcinoma is associated with a grim prognosis in the metastatic state (2). Platinum based chemotherapy is the current gold standard for metastatic disease (3), albeit the fact that median overall survival (OS) ranges between 12 to 15 months (4) and 12.8 to 14 months for patients ineligible for platinum based therapy receiving vinflunine-carboplatin or vinflunine-gemcitabine (5). Options seemed even more limited in the second line setting, with OS rates of 6.9 months for vinflunine (6). Toxicity related adverse events, the fact that only about half of patients are eligible for first line cisplatin (7), together with the poor outcome in the second line setting have emphasized the need for alternative therapeutic regimens for decades.

Currently used checkpoint inhibitors for urothelial carcinoma counteract immune evasion of cancer cells by blocking the interaction between programmed death 1 (PD-1) receptor and its ligands PD-L1 and PD-L2 (8). In Europe, Atezolizumab, Nivolumab, and Pembrolizumab have been approved for second line treatment, while Atezolizumab and Pembrolizumab may also be used in the first line setting, i.e., for patients ineligible for cisplatin based chemotherapy (9–13). Today, the use of checkpoint inhibition in the first line setting is tied to the expression of the transmembrane protein PD-L1 in cancer tissue and the presence of immune cells (14).

In this study we take a first look at real world data and first impressions on all three available substances for the treatment of advanced urothelial carcinoma. Our main goal was to evaluate clinical data on checkpoint inhibition for urothelial cancer patients in a real-world setting.

## Patients And Methods

All patients included in this study had confirmed histopathology of urothelial carcinoma. All patients received intravenous monotherapy with either Atezolizumab, Nivolumab or Pembrolizumab with the approved dosages of 1200 mg q3weeks, 3 mg/kg q2weeks, and 200 mg q3weeks, respectively. Durvalumab and Avelumab were not approved in Europe outside of clinical trials and were not used. Only patients progressing after or during chemotherapy were included. Multiple regimens (≥1) of chemotherapy prior to checkpoint inhibition were allowed. Patients with both, lower and upper tract urothelial carcinoma were included. Patients with adenocarcinoma or sarcomatoid differentiation were excluded.

Routine laboratory values prior to checkpoint inhibitor administration as well as performance-status according to the Eastern Cooperative Oncology Group (ECOG) were recorded (15). The Bellmunt criteria (ECOG performance-status > 0, hemoglobin concentration of less than 10 g per deciliter and presence of liver metastases) were applied for stratification of patients into risk groups (16).

All patients were followed with staging imaging. Metastatic lesions were assessed according to the Response Evaluation Criteria in Solid Tumors (RECIST, version 1.1. (17)). Adverse events in general and immune related adverse events were defined and recorded according to the National Cancer Institute Common Terminology Criteria for Adverse Events (version 4.03.). Immune-related events were counted only once per organ and per patient.

Prospective and ongoing data collection was performed in a prospectively designed, multi-center relational database. This retrospective study was carried out in accordance with the current standard of care according to the recommendations of the European Association of Urology (EAU) guidelines on treatment of metastatic urothelial carcinoma. The protocol and the retrospective analysis of anonymous data were approved by the Ethics Committee of Hanover Medical School, Hanover, Germany. All subjects gave written informed consent in accordance with the Declaration of Helsinki.

The data cutoff for the current analysis was December 12th 2018. For descriptive data presentation, categorical data was shown with absolute numbers and percentages. Continuous variables were presented with either the mean and the standard deviation or the median with range. Progression free (PFS) survival and OS were calculated with the Kaplan-Meier estimation method. R statistical software was used for statistical analysis, figures and tables (18).

## Results

**Figure 1:**
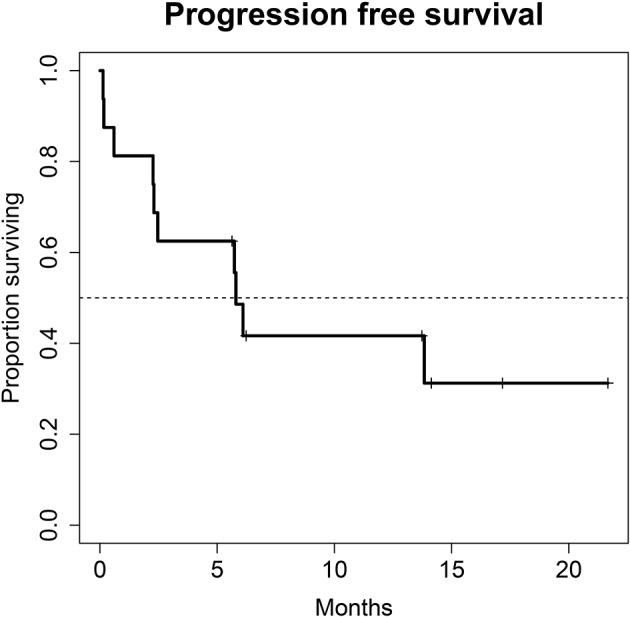
Progression free survival (PFS) for 28 patients under second line therapy with checkpoint inhibitor monotherapy for metastatic urothelial carcinoma. Median PFS was 5.8 months (95% CI, 2.3–NA months).

**Figure 2:**
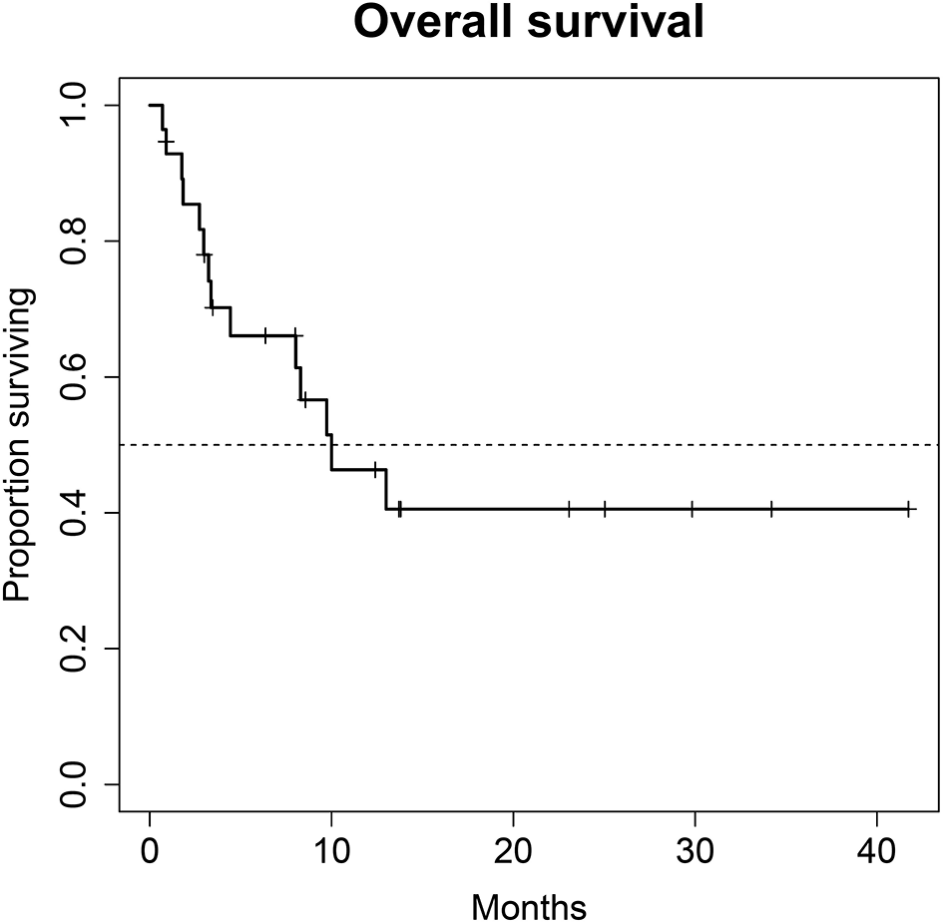
Overall survival (OS) for 28 patients under second line therapy with checkpoint inhibitor monotherapy for metastatic urothelial carcinoma. Median OS for all patients was 10.0 months (95% CI, 8.0–NA months).

**Figure 3:**
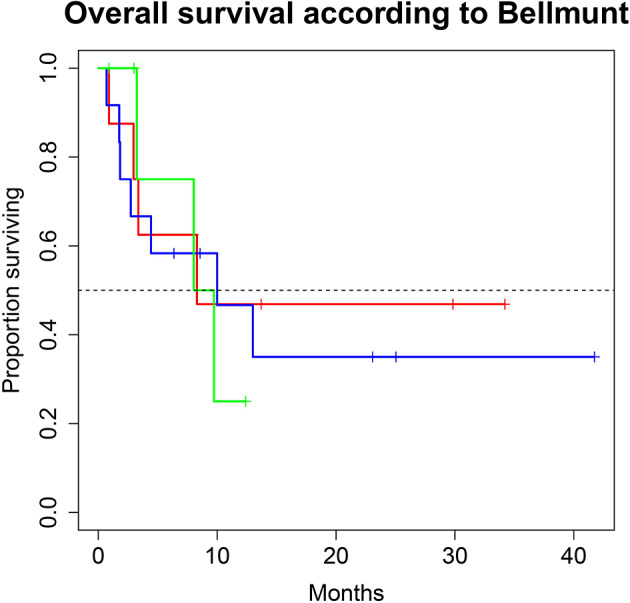
Overall survival (OS) for 28 patients under second line therapy with checkpoint inhibitor monotherapy for metastatic urothelial carcinoma according to Bellmunt criteria [Risk score 0 (green) vs. 1 (blue), vs. ≥2 (red)]. OS did not differ between groups (8.3, 10.0 and 8.9 months; *p* = 0.9).

**Figure 4:**
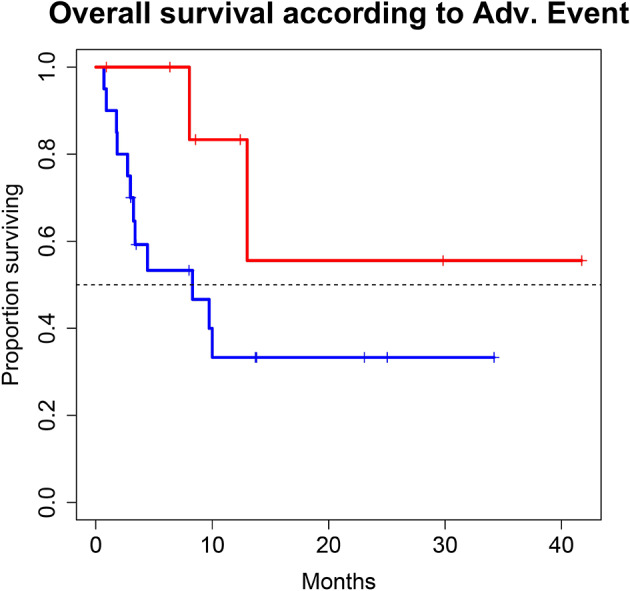
Overall survival (OS) for 28 patients under second line therapy with checkpoint inhibitor monotherapy for metastatic urothelial carcinoma according to occurrence of immune related adverse events [Grade 0-1 (blue) vs. ≥2 (red)]. OS differed in favor for patients with immune related adverse events (8.3 months vs. not reached, *p*-value = 0.1067), however this difference was not statistically significant.

**Figure 5:**
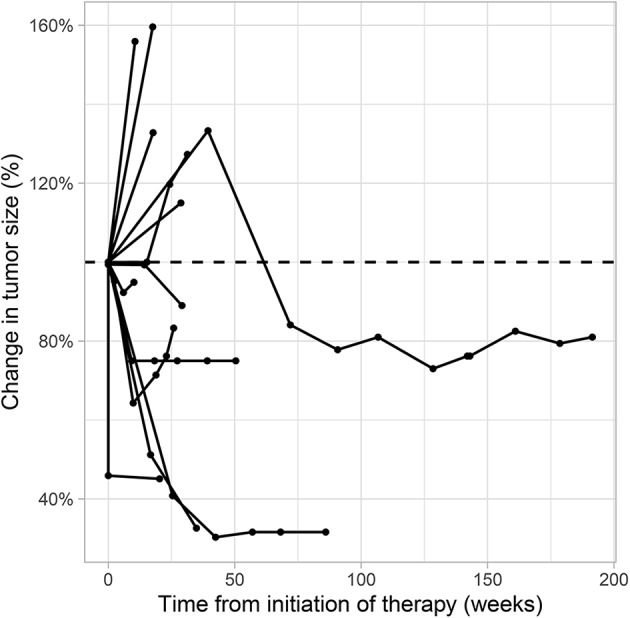
Spider plot showing the percentage of target lesion tumor size change over time for each patient under second line therapy with checkpoint inhibitor monotherapy for metastatic urothelial carcinoma. Radiological data on tumor size change was available for 13 patients who had >1 evaluable cross-sectional imaging. Target lesion size was measured according to the Response Evaluation Criteria in Solid Tumors [RECIST v1.1 (17)].

**Figure 6:**
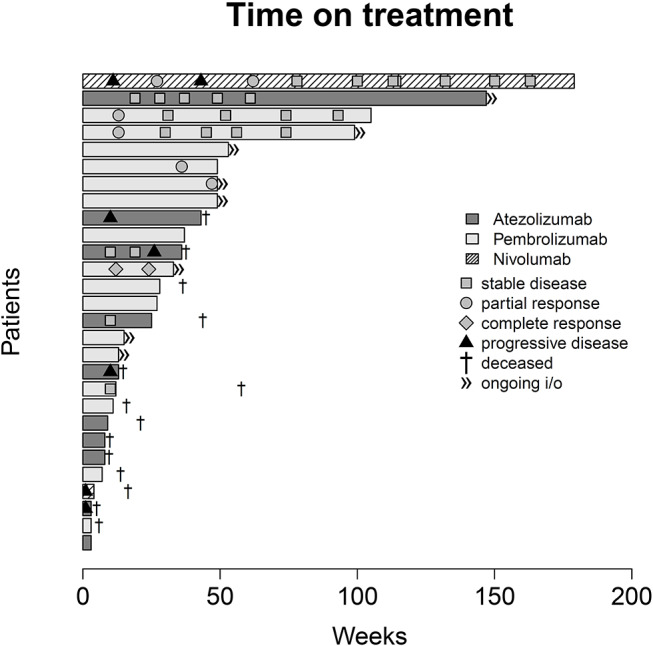
Swimmer plot indicating duration of checkpoint inhibitor monotherapy, overall survival and radiological response according to the Response Evaluation Criteria in Solid Tumors [RECIST v1.1 (17)].

A total of 28 patients from 3 separate institutions were included. Data was collected between 01/2016 and 02/2020. Patient characteristics are summarized in (Table 1). All 28 patients were given checkpoint-inhibition after prior chemotherapy. The number of patients receiving Atezolizumab, Pembrolizumab or Nivolumab were 10 (35.7%), 16 (57.1%), and 2 (7.1%), respectively. Data on PD-L1 status was scarce due to the fact, that all patients presented here were not part of any clinical trial. Duration of follow-up was defined as the time from first administration of the checkpoint-inhibitor to the date of the last clinical visit. The median follow-up was 8.0 (range, 0.7–41.7) months. Median duration of therapy for all patients was 6.05 (range, 0.7–41.8) months. Median PFS was 5.8 (95% CI, 2.3–NA, Figure 1) months. Median OS for all patients was 10.0 (95% CI, 8.0–NA months, Figure 2). OS did not differ between different scores for Bellmunt (16) risk criteria (risk score: 0, 1, ≥2) with estimated OS times of 8.3, 10.0, and 8.9 months (*p* = 0.9, Figure 3). From clinical experience we tend to see good oncological control for patients who develop immune related adverse events. We could demonstrate this difference when comparing patients with and without immune related adverse events: Patients with no event vs. grade ≥2 (8.3 months vs. not reached, *p*-value = 0.1067), however this difference was not statistically significant (Figure 4). At the end of data collection, a total of 8 (28.6%) patients were still under active checkpoint-inhibitory therapy. The overall response rate was 21.4% (6 out of 28 patients; 95% CI, 6.2%−36.6%). The median time to response was 13.1 weeks. The median duration of response was 16.4 weeks. At data cutoff, 5 (83.3%) out of 6 initially responding patients had an ongoing response. Change in target lesion size and RECIST Data are illustrated in (Figures 5, 6). Adverse events were recorded in 20 (71.4%) patients. Higher grade adverse events (≥Grade 3) were present in 11 (39.3%) cases. No therapy related deaths occurred during the observation period. A total of 13 (46.4%) patients displayed adverse events that were considered to be immune related. Higher grade immune-related adverse events (≥Grade 3) were recorded in 6 (21.4%) cases. The most commonly affected organ was the thyroid gland with 21.4% of events (Tables 2, 3).

**Table 1:** Patient characteristics for 28 Patients under checkpoint inhibitor monotherapy with Atezolizumab, Pembrolizumab, or Nivolumab (ECOG = Eastern Cooperative Oncology Group; NA = not available; ^*^ECOG performance-status >0, hemoglobin concentration <10 g/dl, presence of liver metastases (16).

**Patient characteristic**	**Parameter**	**%, Range**
**Patients**	28	100%
Age (median, range)	67.5 yrs.	53–80 yrs.
**Gender**		
Male	19	67.9%
Female	9	32.1%
**Primary tumor**		
urinary bladder	15	53.6%
upper urinary tract	5	17.9%
Unspecified	8	28.6%
**Prior chemotherapy**		
Gemcitabine/Cisplatin	23	82.1%
Gemcitabine/Carboplatin	3	10.7%
Carboplatin/Paclitaxel	2	7.1%
Vinflunine	2	7.1%
**ECOG**		
0	13	46.4%
1	5	17.9%
2	8	28.6%
3	0	0%
4	0	0%
NA	2	7.1%
**Metastases**		
Liver	3	10.7%
Visceral	3	10.7%
Bone	3	10.7%
**Hemoglobin**		
≥10 g/dl	18	64.3%
<10 g/dl	10	35.7%
Number of Bellmunt risk criteria*		
0	8	28.6%
1	12	42.9%
2	6	21.4%
3	0	0%
NA	2	7.1%

**Table 2:** Adverse events.

**CTCAE Grade**	**Any event**	**Immune-related**
Any Grade	20 (71.4%)	13 (46.4%)
Grade ≤ 2	9 (32.1%)	7 (25%)
Grade ≥3	11 (39.3%)	6 (21.4%)

*Summary for patients with total adverse events and with immune-related adverse events. Numbers are shown as total number of afflicted patients per grading interval (Grade ≤ 2 or Grade ≥3) and percentage with regard to the total patient number of n = 28 patients (CTCAE = Common Terminology Criteria of Adverse Events)*.

**Table 3:** Summary of immune-related adverse events.

**Organ**	**Events**	**%**
Colitis	1	3.6%
Skin	3	10.7%
Thyroid	6	21.4%
Liver	3	10.7%
Hypophysis	1	3.6%
Skeletal	1	3.6%
Pancreas	0	0%
Pharynx	0	0%
Renal	4	14.3%
Other	4	14.3%

*Numbers are shown as total number of patients with immune-related adverse events per organ and as percentage of the study population of n = 28. In total, 23 immune-related events in 13 patients were recorded*.

## Discussion

This series of patients does not represent a randomized controlled trial with a defined competitor. Our main point of discussion focuses on the question whether or not real-life treatment of patients, outside of trial associated selection and restrictions, can reproduce the published data on treatment response and tolerability.

Regarding treatment response, our PFS survival almost reached 6 months. In comparison, PFS in the intention to treat population of randomized clinical phase II and III trials of checkpoint inhibition showed a PFS of no longer than 2.1 months in all trials (10, 12, 13, 19). This discrepancy is most likely due to the fact that our study population is still rather small. Also, in this series of real-life data, imaging did not follow the strict 3-monthly intervals as scheduled in the above-mentioned trials, also a very reasonable explanation for the observed PFS. Therefore, progression may have been picked up late, at least in a subgroup of our patients. A systematic comparison of response rates and survival data of the current literature are shown in (Table 4). We were able to achieve a response rate of over 21% over all. Evaluating responses with regard to each of the three substances individually was not feasible from a statistical standpoint considering the low and uneven patient count for each group. Also, the expected variance in response rates in cohorts of 200 to 400 patients (as were evaluated in the above-mentioned trials) is rather high: Response rates from the literature show that only about every 5th patient responds to checkpoint inhibition monotherapy. Our data is consistent with this finding. However, the assumed response rates follow a binominal distribution with rather wide confidence intervals. When assuming an actual response rate of 20%, we calculated that 95% of response results would fall between 15.5% and 24.5% in a cohort of 300 patients. This explains the wide confidence intervals on response rates reported for Atezolizumab, Nivolumab and Pembrolizumab (10, 12, 13, 19). A more representative estimation on response, but only for Atezolizumab, can be extracted from the SAUL trial that comprised *n* = 1004 patients. Unfavorable conditions, such as an ECOG performance status of 2, cerebral metastases or autoimmune disease, among others, were allowed. OS in the intention-to-treat population was 8.7 months (95% CI 7.8–9.9 months), which is comparable with our results. When exclusively looking at patients (*n* = 643) from the SAUL trial who had similar inclusion criteria as in the IMvigor211 trial, median OS improved to 10.0 (95% CI 8.8–11.9) months. ORR was 13% (11–16%) months with a disease control rate of 40% (37–43%) (20).

**Table 4:** Overall response rates (ORR), progression free survival (PFS), overall survival (OS) and severe adverse events (AE, Adverse events according to the common terminology criteria for adverse events, grade ≥3) for patients treated with checkpoint inhibition monotherapy for metastatic urothelial carcinoma in the second-line setting.

**Substance**	**Patients (*n*)**	**Target**	**ORR (%)**	**PFS (month)**	**OS (month)**	**Severe AE (%)**
Atezolizumab*Mvigor210[cohort 2] (10)*	310	PD-L1	15.0%	2.1	7.9	16%
Atezolizumab*IMvigor211* (19)	467	PD-L1	13.4%	2.1	8.6	20%
Nivolumab*Checkmate275 (13)*	270	PD-1	19.6%	2.0	8.73	18%
Pembrolizumab*Keynote045 (12)*	270	PD-1	21.1%	2.1	10.3	15%

*All numbers refer to the intention to treat population. PD-L1 (programmed cell death ligand 1), PD-1 (programmed cell death protein 1)*.

With regard to OS, our real-world analysis reproduced the promising results from prior trials. As seen in the swimmer plot (Figure 4), a few patients had a short duration of treatment and died early. This may be related to the fact that most patients receiving Atezolizumab were included in the expanded access program. Some of these patients had extensive metastatic load, multiple prior regimens of chemotherapy and were given checkpoint inhibition very late in the course of the disease. Taking this into consideration, OS might improve with patients being more and more able to receive checkpoint inhibition earlier on. Gathering real life data on checkpoint inhibition is therefore important.

Regarding the safety of treatment, checkpoint inhibition exhibited a more favorable safety profile than chemotherapy, as could be expected from trials with chemotherapy as a competitor (12, 19). OS differed in favor for patients with immune related events. Albeit the fact, that this difference was not statistically significant, our data support the concept, that the presence of immune related adverse events may correlate to some extent with an increased likelihood of treatment efficacy. The thyroid gland was the most prevalently afflicted organ. Colitis, in contrast to prior trials, was not a major issue in this series. However, we did see events of immune mediated colitis in our cohort of patients with checkpoint inhibition in the first line setting (data not shown).

As a limitation, data quality may not be comparable to data derived from randomized controlled trials: In particular, RECIST evaluation was performed by multiple radiologists from 3 different institutions and imaging did not follow a strict time schedule as is the case in clinical trials. Last, a variety of inclusion and exclusion criteria do not apply in this real-world setting, hence data is less homogenous.

## Conclusion

Our real-world clinical series confirms an objective response for about every fifth patient, promising OS and a low incidence for severe adverse events (≥Grade 3). In total, our experience with checkpoint inhibition monotherapy reflects, and to some extend surpasses, oncological efficacy and safety and is comparable with the experience from randomized trials for these substances.

## Data Availability Statement

The datasets generated for this study are available on reasonable request to the corresponding author.

## Author Contributions

CF: resources, data curation, writing—original draft preparation. JS and PI: validation, data curation. MWK, IP, AM, MAK, and MH: validation. BH and AF: investigation, validation, data curation. C-AK: conceptualization, methodology, formal analysis, writing—review and editing, project administration.

## Conflict of Interest

The authors declare that the research was conducted in the absence of any commercial or financial relationships that could be construed as a potential conflict of interest.
